# Binding of Transcriptional Activator to Silent Loci Causes Their Detachment from the Nuclear Lamina in *Drosophila* Neurons and Salivary Gland Cells

**DOI:** 10.3390/ijms26125793

**Published:** 2025-06-17

**Authors:** Ruslan A. Simonov, Oxana M. Olenkina, Valentina V. Nenasheva, Yuri A. Abramov, Sergey A. Lavrov, Anna A. Fedotova, Yuri Y. Shevelyov

**Affiliations:** 1 Laboratory of Analysis of Gene Regulation, National Research Centre “Kurchatov Institute”, 123182 Moscow, Russia; simonoff.ra@mail.ru (R.A.S.); oxamyth@gmail.com (O.M.O.); abramov75@rambler.ru (Y.A.A.); 2 Laboratory of Molecular Neurogenetics and Innate Immunity, National Research Centre “Kurchatov Institute”, 123182 Moscow, Russia; val-nenasheva@mail.ru; 3 Laboratory of Biochemical Genetics of Animals, National Research Centre “Kurchatov Institute”, 123182 Moscow, Russia; slavrov.defy@gmail.com; 4 Department of Regulation of Genetic Processes, Institute of Gene Biology, Russian Academy of Sciences, 119334 Moscow, Russia; annafedotova@list.ru

**Keywords:** transcriptional activator, nuclear lamina, nuclear periphery, lamina-associated domain, salivary glands, neurons, *Drosophila*

## Abstract

In mammals, the binding of transcriptional activators leads to the repositioning of silent loci from the nuclear periphery to the nuclear interior. However, it remained unknown whether the same mechanism functions in *Drosophila*. Here, using FISH and DamID, we have shown that binding the GAL4 activator to the silent loci causes weakening of their interactions with the nuclear lamina and relocalization inside nuclei in *Drosophila* salivary gland cells and neurons. This mimics the removal from the nuclear periphery of a neuron-specific gene upon its activation in neurons. Salivary gland cells contain polytene chromosomes with mechanical properties, different from chromosomes of diploid cells, while neurons represent predominantly non-dividing cell type. Our results indicate a causal relationship between transcriptional activator binding and changes in the intranuclear position of loci in *Drosophila*. They also point to the similarity in general chromatin dynamics in mammals and *Drosophila*, thus strengthening the role of model organisms in studying genome architecture.

## 1. Introduction

It is well-known that nuclear periphery represents the repressive compartment in the nucleus (reviewed in [1]). Numerous fluorescence in situ hybridization (FISH) data in different organisms indicate that, upon activation, silent loci relocate from the nuclear periphery to the nuclear interior [2,3,4,5,6,7,8,9]. Accordingly, the loss of interactions between the activated loci and the nuclear lamina (NL) was detected in both mammals and *Drosophila* using the DamID approach [10,11,12,13,14,15,16]. Moreover, in mammals, a causal relationship between activator binding and locus repositioning has been established [15,17,18,19,20]. Long-range locus repositioning was shown to occur in cells within a few hours after the addition of the activator [18]. In mammals, artificial locus tethering to the NL requires cells to go through mitosis [21,22]. Moreover, the knockdown of both lamin genes led to loci repositioning in the cycling mammalian cells but had no effect on their re-localization upon cell cycle arrest [23].

However, it remains unknown whether the binding of a transcriptional activator causes the relocalization of a locus from the NL to the nuclear interior in *Drosophila* cells, particularly in non-dividing *Drosophila* cells, such as neurons. It was also unclear whether a locus in *Drosophila* salivary gland polytene chromosomes, which are built from approximately a thousand aligned chromatids (reviewed in [24]) and are, therefore, more rigid, is displaced from the NL upon activator binding. To address these issues, we applied the *UAS* (Upstream Activating Sequence)/GAL4 system [25] in *Drosophila* neurons and salivary gland cells and analyzed the intranuclear position of two loci using FISH and DamID.

## 2. Results

### 2.1. GAL4 Binding to a Silent Locus Leads to Its Repositioning from the NL to the Nuclear Interior in Salivary Gland Cells

**Figure 1:**
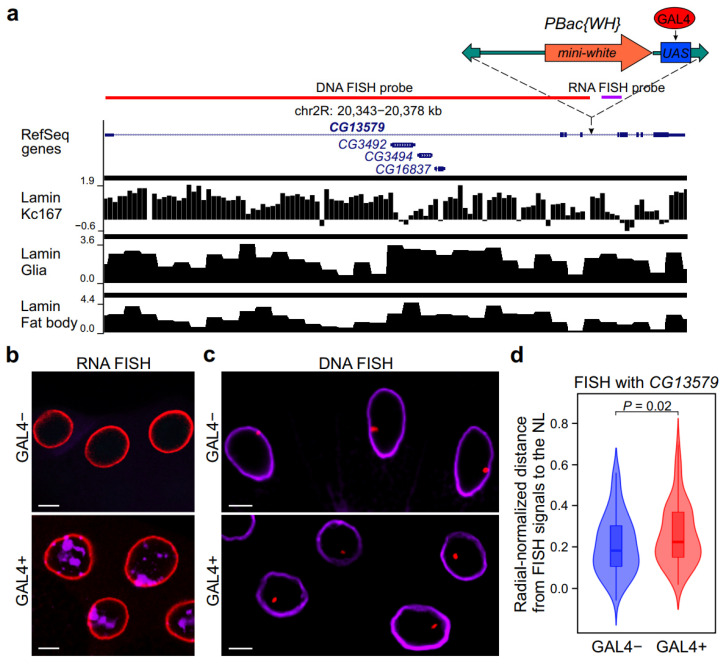
GAL4 binding with the *UAS* element of the *piggyBAC-WH* transposon results in partial redirection from the NL of the transposon-carrying region. (**a**) Structure of *CG13579* gene region, carrying the *piggyBAC-WH* transposon and three other genes. Shown are RefSeq genes, log_2_(Dam-lamin/Dam) profiles in Kc167 cells [28], in glia and fat body [29] with LADs as black rectangles over profiles. *piggyBAC* inverted repeats are shown as green arrows, the *mini-white* gene is marked by orange, the *UAS* element is marked by blue, and the GAL4 activator is indicated by a red oval. The probe for DNA FISH is shown by a red line, and the probe for RNA FISH by a violet line. (**b**) The representative example of RNA FISH signals (violet) in GAL4− and GAL4+ salivary glands with an antisense probe for GAL4-induced transcription downstream from *piggyBAC-WH* transposon. NL is stained with anti-lamin Dm0 antibodies (red). Scale bars = 10 µm. (**c**) The representative example of DNA FISH signals (red) in GAL4− and GAL4+ salivary glands with the probe for the *CG13579* gene. NL is stained with anti-lamin Dm0 antibodies (violet). Scale bars = 10 µm. (**d**) Violin plots showing the distribution of radial-normalized distances between *CG13579* DNA FISH signals and the NL in salivary gland cells from GAL4− (*n* = 101) and GAL4+ (*n* = 128) lines. The *p*-value was calculated with a Mann–Whitney U-test.

We chose salivary gland cells for analysis of intranuclear position because they contain polytene chromosomes, the mechanical properties of which differ from that of interphase chromosomes in diploid cells. Additionally, polytene chromosomes are formed through endoreplication, i.e., without cell division. To examine whether intranuclear localization of silent locus changes upon activator binding, we generated homozygous fly line carrying the *piggyBAC-WH* transposon with 14 *UAS* repeats [26], which was inserted at the *60D* site of the second chromosome (within the intron of *CG13579* gene; Figure 1a), combined with *Act5C::GAL4* transgene on the third chromosome (hereafter GAL4+ line). The control GAL4− line was homozygous for the *piggyBAC-WH* insertion but did not contain the *Act5C::GAL4* transgene.

According to the FlyAtlas database [27], the *CG13579* gene, as well as three other genes located in its large intron, are not expressed in the salivary glands. Lamin-DamID profiles [28,29] indicate that the whole region encompassing the *CG13579* gene represents the lamina-associated domain (LAD) in embryonic Kc167 cell culture, glial cells, and fat body cells (Figure 1a). Therefore, it is tempting to suggest that this region is the LAD in the salivary gland cells as well.

Using RNA FISH, we detected a high level of nascent transcription downstream of *piggyBac-WH* insertion in the salivary glands from the GAL4+ line (Figure 1a,b). This transcription is likely induced by GAL4 binding with the *UAS* element of the transposon because transcripts were not detected in the salivary glands from GAL4- line.

To determine the intranuclear position of the *CG13579* gene in the salivary glands, we performed DNA FISH on GAL4− and GAL4+ lines using a probe corresponding to the *CG13579* gene (Figure 1a,c). NL was stained with anti-lamin Dm0 antibodies. Next, we determined the radial position of the *CG13579* gene in salivary gland nuclei from GAL4− and GAL4+ lines using Imaris software (see Section 4 for details) (Supplementary Table S1). In the salivary glands of the GAL4− line, the localization of half of all *CG13579* FISH signals was at the nuclear periphery (within the peripheral zone of 0.2R width) (Figure 1c,d). However, GAL4 binding to the *UAS* element leads to locus relocalization further away from the NL (Figure 1c,d). These results demonstrate the existence of a causal relationship between activator binding within a LAD and shifting of this LAD from the nuclear periphery to the nuclear interior in *Drosophila* salivary gland cells.

### 2.2. Transcriptional Activation of Neuron-Specific Gene Results in the Loss of Its Contacts with the NL and Relocalization to the Nuclear Interior

Previously, using FISH, we have shown that a cluster consisting of five spermatocyte-specific genes from the *60D* region moves away from the NL upon activation in mature spermatocytes [8]. However, we did not reveal the detachment of spermatocyte-specific genes of this cluster from the NL in mature spermatocytes using DamID [16], which is likely due to the failure of this approach to build a correct lamin profile in the cells that do not divide upon transition from the early to mature spermatocyte stage.

**Figure 2:**
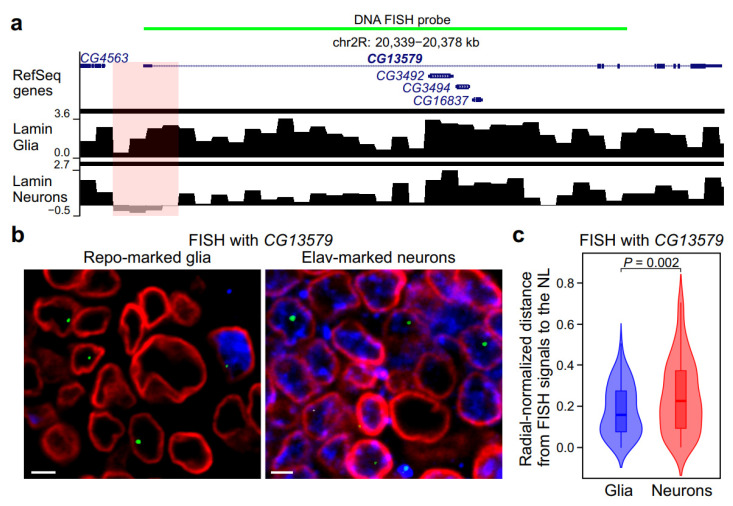
The expression of the *CG13579* gene in neurons and the lack of expression in glial cells correlates with the removal of this locus from the NL in neurons and its positioning at the NL in glial cells. (**a**) The structure of the *CG13579* gene region. Shown are the RefSeq genes, log_2_(Dam-lamin/Dam) profiles in glia and neurons [29], with LADs as black rectangles over profiles. The probe for the DNA FISH is shown in green. The pink semiconfluent rectangle indicates the dip in the lamin profile in neurons but not in the glial cells. (**b**) The representative example of the DNA FISH signals (green) with the *CG13579* probe in Repo-marked glia (**left panel**) and Elav-marked neurons (**right panel**). NL is stained with anti-lamin Dm0 antibodies (red). The brain is stained with anti-Repo (**left panel**) or anti-Elav (**right panel**) antibodies (blue). Scale bars = 2 µm. (**c**) Violin plots showing the distribution of radial-normalized distances between the *CG13579* FISH signals and the NL in Repo-marked glial cells (*n* = 150) and Elav-marked neurons (*n* = 150). The *p*-value was calculated with a Mann–Whitney U-test.

To explore whether we are able to detect the detachment of activated loci from the NL by both FISH and DamID in the non-dividing cells such as neurons, we analyzed the radial position within the nucleus of the *CG13579* gene, which is expressed in neurons and is not expressed in glial cells [30]. Consistent with the idea that loci should loosen contacts with the NL upon activation, the *CG13579* gene has a dip in the lamin-DamID profile around the promoter in the neurons but not in the glia (Figure 2a; lamin-DamID profiles in neurons and glial cells were taken from [29]). We performed FISH with the hybridization probe covering the *CG13579* gene (Figure 2a) in the Elav-positive neurons and Repo-positive glia from third instar larvae (Figure 2b) and counted radial-normalized distances between FISH signals and the NL (Supplementary Table S1). We found that these distances are, on average, larger in neurons than in glial cells (Figure 2c), thus confirming the correlation between locus expression and positioning in the nuclear interior.

### 2.3. In Neurons, GAL4 Binding Within a LAD Results in Local Weakening of Interactions with the NL Around the Binding Site

**Figure 3:**
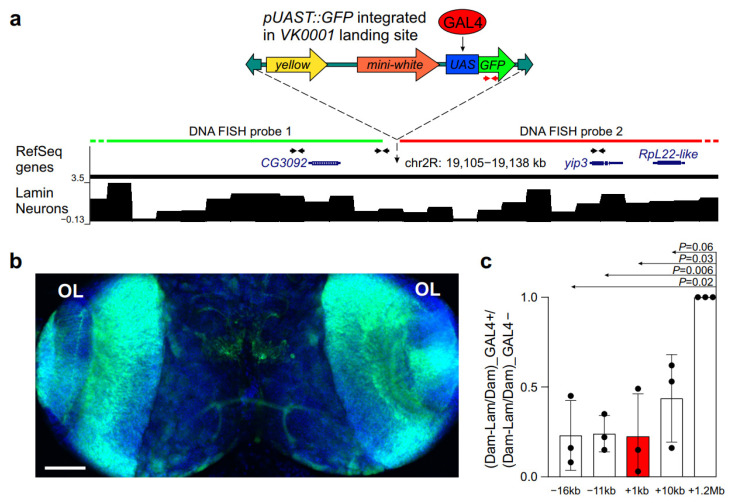
GAL4 binding within a LAD in neurons results in the weakening of interactions with the NL. (**a**) Structure of the *59D* region, carrying *pUAST::GFP* transgene. Shown are the RefSeq genes, log_2_(Dam-lamin/Dam) profile in neurons [29], with LADs as black rectangles over the profile. The *piggyBac* inverted repeats are shown as green arrows; the *mini-white* gene is marked by orange, the *yellow* gene is marked by yellow, the *UAS* element is marked by blue, the GFP is marked by green, and the GAL4 activator is indicated by red. The transgenic markers are not shown to scale. The amplicons for qPCR analysis are shown by red or black arrowheads. The probes for DNA FISH are shown by green and red lines. (**b**) GFP fluorescence (green) and DAPI staining (blue) of *UAS::GFP; elav::GAL4* larval brain. OL—optic lobes. Scale bar = 70 µm. (**c**) The results of qPCR analysis of the degree of Dam- and Dam-lamin-methylation in the amplicon located at ~1-kb distance from the GAL4 binding site (red bar) and at three other amplicons (white bars) located at different distances from the transgene insertion site at the *59D3* region. The GAL4+/GAL4− ratios for each amplicon are normalized to that of the control amplicon located ~1.2 Mb away from the transgene.

Next, we explored by the DamID approach whether GAL4 binding leads to the weakening of interactions between a locus located within a LAD and the NL in neurons. To this end, we generated a fly line carrying the *UAS::GFP* transgene at the *59D3* site [31] using *ϕC31*-mediated site-specific integration of this transgene via the *pUASTattB* vector [32]. According to our earlier published data [29], the *piggyBac-yellow^+^-attP* landing site of the VK00001 insertion is mapped within a LAD in *Drosophila* neurons (Figure 3a). We then genetically constructed a fly line containing the *UAS::GFP* transgene on the second chromosome and the *elav::GAL4* transgene on the third chromosome. As a control line, we employed the *UAS::GFP* transgene without *elav::GAL4*. Microscopy imaging demonstrates that GFP fluorescence in the brain from the *UAS::GFP; elav::GAL4* larvae are mainly found in the optic lobes of the brain (Figure 3b), thus indicating that the GAL4 activator is bound with the *UAS::GFP* transgene in neurons.

Further, we genetically combined these constructs with the constructs for performing lamin-DamID (we employed the “intein” DamID system, which was shown to work well in the *Drosophila* brain [33]). After in vivo Dam-lamin (or Dam-only) methylation, we isolated genomic DNA from the central brain of larvae males and carried out PCR-amplification of methylated fragments (Supplementary Figure S1) according to previously published protocol [34]. The degree of methylation of Dam-lamin and Dam-only was assessed by qPCR in the GFP region adjacent to the GAL4 binding site, as well as in three regions located ~16 kb and ~11 kb upstream or ~12 kb downstream from the GAL4 binding site (Figure 3a). As a negative control, we used LAD from the *60D* region located at ~1.2 Mb distance from the GAL4 binding site. One can see that the binding of GAL4 to the *UAS* element results in the weakening of interactions between the *GFP* region and the NL, as compared to their interactions without GAL4 binding (Figure 3c, red column). The other analyzed sites also exhibited weakened binding with the NL, as compared to the *60D* region. We conclude that the binding of transcriptional activator causes the detachment from the NL of the analyzed locus together with the surrounding regions in *Drosophila* neurons.

### 2.4. In Neurons, GAL4 Binding to a Silent Locus Leads to the Locus Repositioning from the NL to the Nuclear Interior

**Figure 4:**
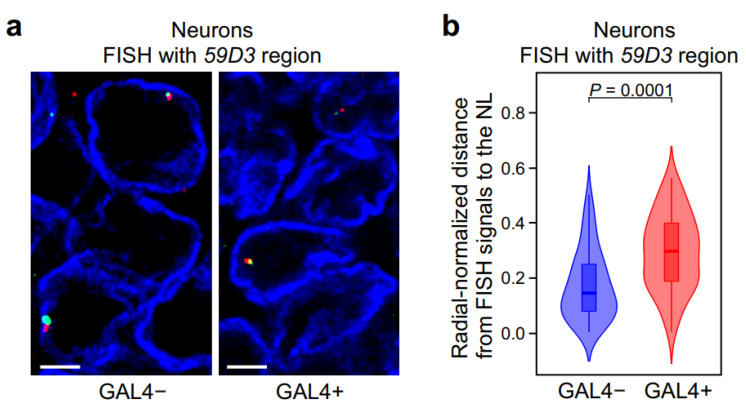
GAL4 binding within LAD results in the removal of this LAD from the NL in neurons. (**a**) The representative examples of DNA FISH signals (red and green) with *59D3* probes in neurons in GAL4− (left panel) and GAL4+ (right panel) lines. NL is stained with anti-lamin Dm0 antibodies (blue). Scale bars = 2 µm. (**b**) Violin plots showing the distribution of radial-normalized distances between *59D3* FISH signals (green) and the NL in GAL4− (*n* = 100) and GAL4+ (*n* = 100) neurons. The *p*-value was calculated with a Mann–Whitney U-test.

We then analyzed by FISH (Figure 4a) whether the LAD from the *59D3* region is shifted from the nuclear periphery upon GAL4 binding. FISH was performed with larval central brain from fly lines homozygous for the *UAS::GFP*; *elav::GAL4* transgenes or for only the *UAS::GFP* transgene (as a control) using hybridization probes, labeled with green and red fluorescent dyes, to the regions flanking the *UAS::GFP* transgene (Figure 3a). We used two adjacent probes, which should be located in close proximity to each other in the nuclear space, for more reliable identification of hybridization signals. After confocal microscopy imaging, we counted radial-normalized distances between green FISH signals and the NL in the neurons using Imaris software (Supplementary Table S1). It should be noted that, upon GAL4 binding, the green fluorescence of GFP did not interfere with the detection of green FISH signals since GFP was inactivated by heating during the FISH procedure. Figure 4b demonstrates that, in neurons, binding of GAL4 to the *UAS* element results in the notable relocalization of the region carrying this *UAS* element to more interior position. Therefore, we revealed the causal relationship between transcriptional activator binding to a region and shifting of this region to the nuclear interior in predominantly non-dividing *Drosophila* cells, such as neurons.

## 3. Discussion

Previous studies on the mammalian cell lines have clearly shown that the binding of transcriptional activator with a locus is sufficient for its relocalization [15,17,18,19,20]. However, such data were absent in *Drosophila*. Given that the median size of *Drosophila* LADs is five times smaller than that of mammals [28], it was interesting to explore whether various *Drosophila* cell types exhibit the same chromatin dynamics as mammals. Here, using DamID and FISH, we found that: (i) the binding of transcriptional activator with silent loci causes their detachment from the NL and redirection to the nuclear interior in *Drosophila* tissues; (ii) this mechanism is also applicable for polytene chromosomes which are more rigid in bending than chromosomes from diploid cells; (iii) the same mechanism operates in mainly non-dividing cells such as neurons. Therefore, the binding of transcriptional activator to a silent locus causes the removal of this locus from the NL in *Drosophila*. Moreover, this mechanism seems to be universal since it is active in various organisms and chromosomes, as well as in both dividing and non-dividing cell types.

The current understanding of the relocalization process is that the myosin motor can move chromatin fiber either toward or away from the nuclear envelope along the nuclear actin filaments. The first indications that nuclear actin and myosin are involved in the long-range directional movement of the loci in response to activator binding were obtained nearly two decades ago on mammalian cell lines [18,35]. More recently, nuclear actin and myosin were shown to be the main actors in the directed motion towards the nuclear periphery of heterochromatic regions containing double-strand breaks [36,37]. Furthermore, nuclear actin and myosin were found to be required to keep the transcribed loci within the transcription factories [38]. In yeast, the relocalization of the *INO1* locus to nuclear pores upon its activation was also dependent on nuclear actin and myosin [39] (reviewed in [40]). In this case, the relocalization was initiated by binding the transcriptional activator to the promoter of the inactive gene, which resulted in the recruitment of myosin motor [39].

Our findings in *Drosophila*, combined with the data from other organisms, point to the model that the nuclear myosin motor, recruited to silent loci by transcriptional activators, drives the relocalization of these loci from the nuclear periphery along actin filaments to transcription factories located in the nuclear interior.

## 4. Materials and Methods

### 4.1. Plasmid Construction

To generate the *pUASTattB-GFP* construct, the *GFP* sequence was excised by NheI and NotI from *pAct::GFP* plasmid and cloned into SpeI and NotI sites of *pBlueScript SK II* vector (Stratagene). Next, the EcoRI-NotI fragment from this plasmid containing *GFP* was recloned into the EcoRI and NotI sites of the *pUASTattB* vector [32].

### 4.2. Fly Stocks and Handling

Fly stocks were maintained under standard conditions at 25 °C.

To perform GAL4 binding with the *UAS* element from *PBac{WH}*, we combined *PBac{WH}CG13579^f00432^* transposon at the *60D* site (#18327, Bloomington Drosophila Stock Centre) with the *Act5C::GAL4* transgene (driver line #3954, Bloomington Drosophila Stock Centre) using standard genetic crosses.

A transgenic strain carrying *pUASTattB-GFP* was generated by *ϕC31*-mediated site-specific integration at the *59D* site on chromosome *2* in the y^1^ M{vas-int.Dm}ZH-2A w*; PBac{y+-attP-3B}VK00001 line (#24861, Bloomington Drosophila Stock Centre) as was previously described [32].

To generate fly lines for performing lamin-DamID in the larval brain by “intein” DamID system [33], we used standard genetic crosses to combine Dam or Dam-lamin constructs (lines #65429 and #65430, Bloomington Drosophila Stock Centre) with the *UAS::GFP* transgene (this study) and with the *elav::GAL4* transgene (driver line #8760, Bloomington Drosophila Stock Centre). As a result, four lines were obtained: (1) *Dam/UAS::GFP*; *elav::GAL4/+* (2) *Dam-lamin/UAS::GFP*; *elav::GAL4/+* (3) *Dam/UAS::GFP*; *+/+* (4) *Dam-lamin/UAS::GFP*; *+/+*.

To generate lines for performing FISH in the *59D3* region, we used standard genetic crosses to obtain *UAS::GFP/UAS::GFP*; *elav::GAL4/+* and *UAS::GFP/UAS::GFP*; *+/+* lines.

### 4.3. Preparation of Probes for RNA and DNA FISH

To obtain the probe for the DNA FISH with the *CG13579* gene, six fragments with a total length of ~30 kb encompassing the *CG13579* gene (Figure 1a and Figure 2a) were PCR-amplified using primers indicated in Supplementary Table S2. To obtain probes 1 and 2 for DNA FISH with *59D3* region indicated in Figure 3a, five fragments with a total length of ~27 kb for probe 1 or with a total length of ~25 kb for probe 2 were PCR-amplified using primers indicated in Supplementary Table S2. The fragments were labeled by digoxigenin(DIG)-dUTP (Invitrogen, Waltham, MA, USA), by AlexaFluor 546-14-dUTP (Invitrogen), or by AlexaFluor 488-5-dUTP (Invitrogen) using the Klenow fragment and random primers.

To obtain a probe for detection of *PBac{WH},* downstream transcription using RNA FISH, a 0.8-kb fragment of *CG13579* gene (Figure 1a) was PCR-amplified using primers indicated in Supplementary Table S2. As a result, the promoter region for T7-RNA polymerase was attached at its 3′ end. This PCR fragment was used as a template for antisense RNA synthesis with the incorporation of DIG-UTP using DIG RNA Labeling Mix (Roche, Switzerland).

### 4.4. RNA FISH with Immunostaining

RNA FISH on salivary glands was performed essentially as described in [41]. Briefly, formaldehyde-fixed salivary glands were hybridized with the DIG-labeled antisense RNA probe using tyramide signal amplification. After hybridization, salivary glands were incubated with anti-DIG antibodies conjugated with horseradish peroxidase (1:500; Roche). Then, the salivary glands were incubated with a solution of tyramide-Cy5 (Syntol, Russia) in PBT and H_2_O_2_. Finally, the NL was stained with mouse anti-lamin Dm0 antibodies (ADL84, 1:500, [42]), followed by Alexa Fluor 546-conjugated goat anti-mouse IgG secondary antibodies (Invitrogen), and DNA was counterstained with DAPI.

### 4.5. DNA FISH with Immunostaining

FISH with larval salivary glands or central brain was performed as was described previously [43]. As the primary antibodies, we used sheep polyclonal anti-DIG-Rhodamine (1:60, Roche), rabbit polyclonal anti-lamin Dm0 (1:500, [44]), or mouse anti-lamin Dm0 (ADL84, 1:500, [42]), mouse monoclonal anti-Elav (9F8A9, 1:300, DSHB) and mouse monoclonal anti-Repo (8D12, 1:300, DSHB) antibodies. As the secondary, we used Alexa Fluor 546-conjugated or 647-conjugated goat anti-rabbit IgG (Invitrogen) and Alexa Fluor 647-conjugated goat anti-mouse IgG (Invitrogen) antibodies.

### 4.6. Intranuclear Loci Position Quantification

Three-dimensional image stacks were recorded with a confocal LSM 710 laser scanning microscope (Zeiss, Jena, Germany). Optical sections at 0.35–0.4 μm intervals along the *Z*-axis were captured. Images were processed and analyzed using Imaris 7.4.2 software (Bitplane AG, Schlieren, Switzerland) with a blind experimental setup. Determination of the shortest distance between the FISH signal and the nuclear envelope, stained with antibodies to lamin Dm0, was carried out as was described previously [45]. Briefly, the NL of the individual nucleus was manually outlined in confocal layers, after which the surface of the nucleus was automatically reconstructed. Next, the shortest distance between the FISH signal and the nuclear surface was measured using a “measuring point” instrument. Data were obtained in two or three replicates with 50–78 FISH signals per replicate (Supplementary Table S1). Distances were normalized to the nuclei radii, which were calculated from the volumes of reconstructed nuclei on the assumption that they have a spherical form.

### 4.7. DamID Procedure in Brains

The central brain was isolated in the cold PBS from ~150–200 third instar larvae males of the indicated genotypes (in three biological replicates). Further isolation of genomic DNA amplification of the Dam-only or Dam-lamin-methylated genomic fragments was performed according to [34]. 18 cycles of PCR amplification (1 min at 94 °C, 1 min at 65 °C, 2 min at 68 °C) was applied for DNA samples. After PCR amplification, the DNA samples were purified using a PCR-purification kit (Evrogen) and analyzed using qPCR with the primer pairs indicated in Supplementary Table S2. Data for the four amplicons from the *59D3* region were normalized to that of the *60D* amplicon.

## Data Availability

The original contributions presented in this study are included in the article/Supplementary Material. Further inquiries can be directed to the corresponding author.

## Supplementary Materials

The following supporting information can be downloaded at: https://www.mdpi.com/article/10.3390/ijms26125793/s1.
