# Supplementary material for: Binding of Transcriptional Activator to Silent Loci Causes Their Detachment from the Nuclear Lamina in Drosophila Neurons and Salivary Gland Cells
Source: Int J Mol Sci. 2025 Jun 17;26(12):5793. doi: 10.3390/ijms26125793 (PMC12192806; doi:10.3390/ijms26125793)
Supplement: Supplementary file 1 [file ijms-26-05793-s001.zip › Supplementary Figure S1.pdf]

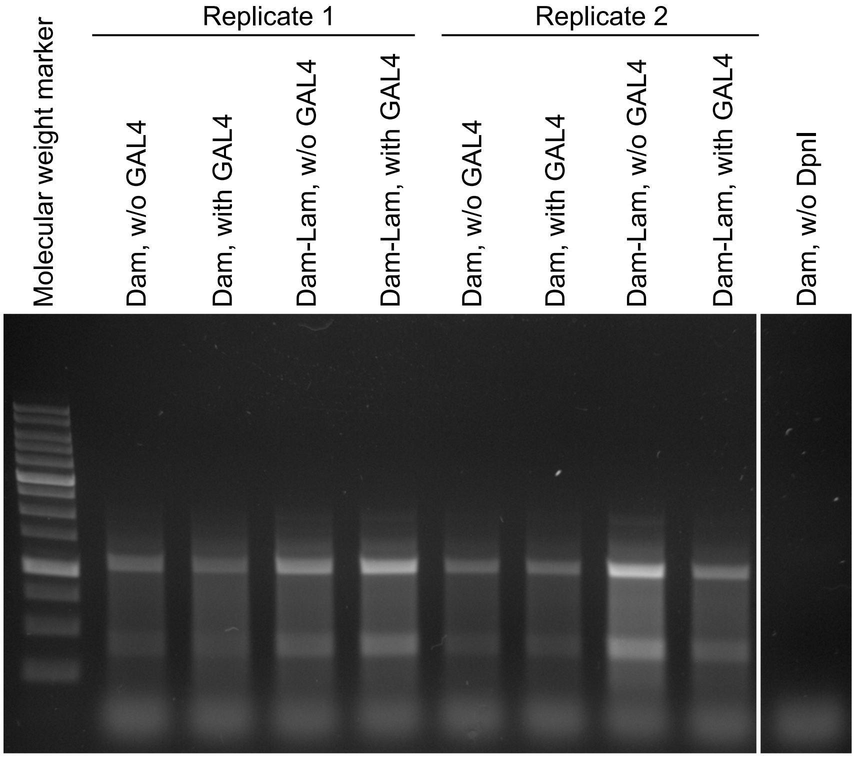


**Figure S1.** Lamin-DamID in larval brain is highly specific. Gel electrophoresis of DNA after PCR-amplification of the methylated genome fragments. The characteristic smear of amplified fragments is much less pronounced in the Dam sample treated without DpnI digestion.
